# Verwendung von Fluoriden zur Kariesprävention

**DOI:** 10.1007/s00103-021-03347-4

**Published:** 2021-06-11

**Authors:** Ulrich Schiffner

**Affiliations:** Zentrum für Zahn‑, Mund- und Kieferheilkunde, Poliklinik für Parodontologie, Präventive Zahnmedizin und Zahnerhaltung, Universitätsklinikum Hamburg-Eppendorf, Martinistr. 52, 20246 Hamburg, Deutschland

**Keywords:** Kariesprävention, Fluorid, Wirkungsmechanismen, Fluoridnebeneffekte, Altersgruppen, Caries prevention, Fluoride, Mechanisms of action, Fluoride side effects, Age groups

## Abstract

Seit wenigen Jahrzehnten ist in Deutschland ein Rückgang der Karieslast in allen Altersgruppen feststellbar. Hierfür wird die regelmäßige Anwendung von Fluoridpräparaten als wesentliche Ursache beschrieben. Es gilt als gesichert, dass für den Rückgang der Kariesprävalenz und Karieserfahrung die lokale Fluoridapplikation, insbesondere durch fluoridhaltige Zahnpasten und Fluoridlacke, auf die Zahnoberflächen in der Mundhöhle verantwortlich ist. Für die klinische Gesamtwirkung wurden einzelne Wirkungsmechanismen wie die Bildung einer Fluoridspeicherschicht, die Remineralisation sowie Effekte auf die bakterielle Plaque bestimmt. Diese Einzeleffekte können an Schmelz und Wurzeldentin bei Patienten jeden Alters wirksam werden. Dabei besteht eine Dosis-Wirkungs-Beziehung zwischen der Fluoridkonzentration in den angewendeten Präparaten und der durchschnittlich erzielten Kariesreduktion.

Es bestehen keine allgemeintoxikologischen Bedenken gegenüber der lokalen Fluoridanwendung. Fluoridzahnpasten sollen ab Durchbruch des ersten Milchzahns verwendet werden. Die Menge der verwendeten Zahnpasta ist auf die empfohlenen Volumina zu begrenzen, um die Entstehung von Schmelzfluorosen zu vermeiden. Die professionell durchgeführte Applikation von hochkonzentrierten Fluoridlacken weist neben einer hohen karieshemmenden Effektivität auch bei erhöhtem Kariesrisiko und bei bereits vorhandenen Demineralisationen besondere Vorteile auf. Dies trifft auch für die Anwendung am Wurzeldentin zu, wo durch Verwendung hochkonzentrierter Fluoridpräparate signifikante primär- und sekundärpräventive Effekte nachgewiesen wurden.

## Abstract (en)

For a few decades, a decrease in the caries burden in all age groups has been observed in Germany. The regular use of fluoride preparations is described as the main reason for the caries decline. It is well established that topical fluoride application directly to the tooth surfaces in the oral cavity, in particular fluoride-containing toothpastes and fluoride varnishes, is responsible for the decline. Several mechanisms of action, such as the formation of a calcium fluoride layer, remineralization, and the effects on bacterial plaque, were determined to be responsible for the overall clinical effect. All these mechanisms can be effective on enamel and root dentin in patients of all ages. There is a dose–response relationship between the fluoride concentration in the applied preparations and the average caries reduction achieved.

There are no general toxicological concerns about topical fluoride application. Fluoride toothpastes should be used from the eruption of the first deciduous tooth. The amount of toothpaste used should be limited to the recommended volumes to avoid the development of enamel fluorosis. The professionally performed application of highly concentrated fluoride varnishes, in addition to having a high caries-inhibiting effectiveness, also exhibits advantages in cases of increased caries risk and existing demineralization. This is also the case for the application on root dentin, where significant primary and secondary preventive effects have been demonstrated by application of highly concentrated fluoride preparations.

## Einleitung

Die Kariesprävalenz und die mittlere Karieserfahrung sind in Deutschland sowohl bei Kindern und Jugendlichen als auch bei der erwachsenen Bevölkerung seit einigen Jahrzehnten deutlich zurückgegangen. Diese für die Zahnkronen des bleibenden Gebisses aufgezeigten Erfolge werden zum großen Teil auf die breite Anwendung fluoridhaltiger Präparate in der Mundhöhle zurückgeführt. Im Milchgebiss und bei frei liegenden Zahnwurzeln sind diese Erfolge weniger ausgeprägt. Daher sind für Kleinkinder wie auch für andere Personengruppen mit erhöhtem Kariesrisiko zusätzliche Präventionsmaßnahmen erforderlich. Da die kariespräventive Wirksamkeit lokaler Fluoridierungsmaßnahmen nachgewiesen ist, wird intensivierten Fluoridanwendungen zukünftig auch eine bedeutende Rolle bei der Kariesprävention in Risikogruppen zukommen. Mit diesem Beitrag sollen die Einzelmechanismen erläutert werden, mit denen Fluorid die klinisch dokumentierten Kariesreduktionsraten ermöglicht, und es sollen zugleich Wege für eine bessere Kariesprävention in den bislang noch vorhandenen Gruppen mit erhöhtem Kariesrisiko durch Verwendung von Fluorid aufgezeigt werden.

## Präventionserfolge mit Fluorid

**Figure 1:**
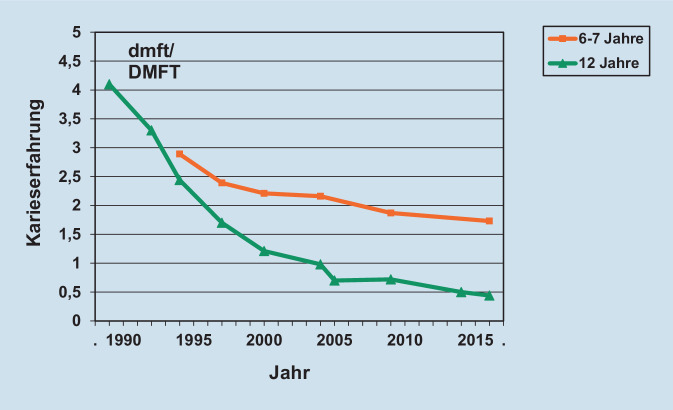

Die Karieslast ist in Deutschland seit einigen Jahrzehnten in allen Altersgruppen rückläufig (Abb. 1). Besonders hoch ist der Rückgang der Kariesprävalenz und Karieserfahrung bei Kindern und Jugendlichen [1]. So kann im bleibenden Gebiss bei 12-jährigen Kindern innerhalb von 25 Jahren ein Rückgang der Kariesprävalenz von 87,6 % auf 17,7 % beobachtet werden (alte Bundesländer) und die mittlere Karieserfahrung (DMFT-Wert, Summe der kariösen („decayed“), kariesbedingt fehlenden („missing“) und aufgrund von Karies gefüllten („filled“) Zähne) hat sich von 4,1 auf 0,4 verringert [1]. In ähnlicher Weise konnte die Karieslast international in vielen Industrieländern reduziert werden.

**Figure 2:**
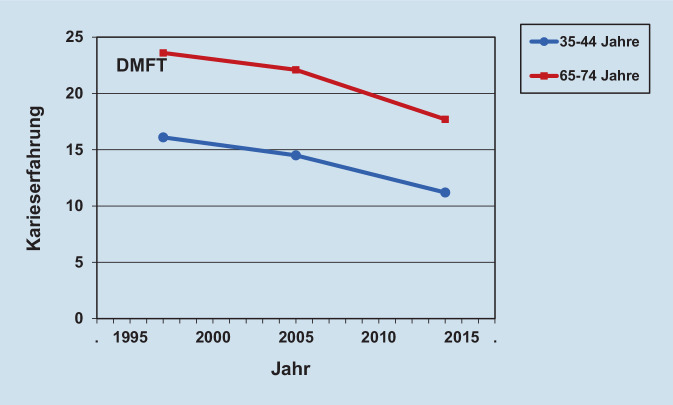

Inzwischen sind die Präventionserfolge nicht mehr nur auf die Altersgruppen der Kinder und Jugendlichen beschränkt. Vielmehr konnte mit der Fünften Deutschen Mundgesundheitsstudie (DMS V) auch bei Erwachsenen im Alter von 35 bis 44 Jahren ein starker Rückgang der Karieserfahrung um ca. ein Drittel dokumentiert werden [1] (Abb. 2). In gleicher Weise konnten auch bei Senioren im Alter von 65 bis 74 Jahren große Verbesserungen der Mundgesundheit ermittelt werden. Insbesondere konnte die Anzahl kariesbedingt extrahierter Zähne um 6,5 Zähne reduziert werden, ohne dass die jetzt vermehrt noch vorhandenen Zähne Karies aufwiesen [1]. Offensichtlich gelingt es also über viele Altersgruppen, die Zähne durch geeignete Präventionsmaßnahmen gesund zu erhalten.

Eine Vielzahl wissenschaftlicher Studien, darauf basierender Reviews und Metaanalysen kommt zu der Schlussfolgerung, dass ein zentrales Element der Kariesprävention, auf dem dieser Rückgang der Kariesprävalenz und Karieserfahrung basiert, die regelmäßige lokale Verwendung von Fluoridpräparaten in der Mundhöhle ist. Insbesondere die breite Verfügbarkeit von Fluorid in Zahnpasten gilt als bedeutende Ursache für diese Entwicklung [2]. Der wesentliche Einfluss regelmäßiger Fluoridapplikationen mittels Zahnpasten auf den Rückgang der Karieslast ist mit hohem wissenschaftlichen Evidenzgrad abgesichert [3, 4]. Für die Kariesreduktion besteht ein direkter, statistisch signifikanter Zusammenhang zur aufgebrachten Fluoridkonzentration: Höher konzentrierte Fluoridzubereitungen erzielen höhere Kariesreduktionen [3, 5].

Im Gegensatz zu den skizzierten deutlichen Erfolgen der Kariesprävention existieren aber auch Alters- und Patientengruppen, bei denen der Rückgang der Kariesprävalenz und Karieserfahrung verhaltener ausfällt. Hierzu zählen Kleinkinder und Kinder, bei denen im Milchgebiss zu früh zu viele kariöse Defekte auftreten und bei denen in einigen Bundesländern sogar eine Stagnation der Karieslast registriert wird ([6]; Abb. 1). Selbst in der Langzeitbetrachtung ist der Rückgang der Kariesprävalenz im Milchgebiss bei 6‑/7-jährigen Kindern nur etwa halb so hoch wie der im bleibenden Gebiss bei 12-jährigen Kindern.

Eine weitere Patientengruppe mit besonderem Kariesrisiko stellen Personen mit frei liegenden Zahnwurzeln dar. Dies sind überwiegend ältere Patienten. Mit der höheren Anzahl eigener Zähne steigt in der älteren Population daher das Risiko von Wurzelkaries und bereits jetzt kann eine Zunahme der Anzahl kariöser Zahnwurzeln beobachtet werden [7].

Bei der Etablierung von Konzepten, um auch in diesen Gruppen zu deutlicheren Erfolgen der Kariesprävention zu kommen, wird die Fluoridanwendung eine bedeutende Rolle spielen. Es sei jedoch betont, dass erfolgreiche Kariesprophylaxe auf einem Gesamtkonzept aller als wirkungsvoll erwiesener Strategien der Kariesprävention basiert. Das bedeutet neben der lokalen Fluoridapplikation die Umsetzung adäquater Mundhygienemaßnahmen zur Reduktion des kariogenen Biofilms sowie eine angemessene Ernährungsweise zur Verringerung der Zufuhr niedermolekularer Kohlenhydrate, in erster Linie von Saccharose [8].

## Wirkungsmechanismen lokal applizierter Fluoride

Über viele Jahre wurde über die Bedeutung der sogenannten systemischen gegenüber der lokalen Fluoridapplikation diskutiert [9]. Unter der lokalen Applikation wird die Anwendung von Fluoridpräparaten wie Zahnpasten, Mundspüllösungen, Gelen oder Lacken direkt auf dem Zahn in der Mundhöhle verstanden, während die systemische Applikation die Aufnahme von Fluorid über Trinkwasser, Tabletten oder Speisesalz und deren Effekt auf die sich noch vor dem Zahndurchbruch in der Entwicklung befindenden Zähne zum Inhalt hat. Inzwischen ist es wissenschaftlich gesichert, dass Fluorid lokal an der Zahnoberfläche karieshemmend wirkt [10, 11]. So werden auch die früher angenommenen Erfolge der „systemischen“ Fluoridgabe heute als Folge des mit der Aufnahme verbunden Kontaktes zu den sich bereits in der Mundhöhle befindenden Zähnen angesehen [9, 10, 12]. Die Einnahme von fluoridangereichertem Speisesalz hat insbesondere dann kariespräventive Effekte, wenn lokale Fluoridierungsmaßnahmen wie die Verwendung fluoridhaltiger Zahnpasten nicht hinreichend umgesetzt werden.

Fluorid erzielt die karieshemmende Wirkung über die im Folgenden erläuterten Mechanismen, die in der Mundhöhle zumeist parallel zueinander an den Zahnoberflächen und in der bakteriellen Plaque ablaufen (Tab. 1).

**Table 1:** 

Ort und Effekt	Bedeutung
*Zahnhartsubstanzen*	
Kalziumfluoriddeckschicht	Fluoridspeicher, stellt Fluorid bedarfsgerecht zur Verfügung
Förderung der Remineralisation	Einfluss auf das dynamische Lösungsgleichgewicht an der Zahnoberfläche zugunsten intakter Mineralstrukturen
Aufnahme von Fluorid in das Hydroxylapatitkristallgitter	Entstehen eines schwerer säurelöslichen Minerals; erhöhte Aufnahme bei Fehlstellen im Kristallgitter (Demineralisationen)
*Biofilm*	
Hemmung der bakteriellen Glykolyse	Reduktion der Säureentstehung aus bakteriellem Kohlenhydratabbau
Besetzen von Anheftungsstellen der Bakterien am Pellikel	Verzögerung der Plaquebildung
Förderung einer bakteriellen Symbiose	Hemmender Einfluss auf die Entstehung eines dysbiotischen, kariogenen Biofilms

### Fluoridhaltige Deckschicht auf der Schmelzoberfläche

Ein bedeutender Fluoridwirkungsmechanismus besteht in der Bildung einer Kalziumfluoriddeckschicht auf der Zahnoberfläche. Hierbei reagiert das zugeführte Fluorid mit Kalziumionen des Zahnes. Es entsteht Kalziumfluorid bzw. ein kalziumfluoridähnliches Material. Leicht saure pH-Werte fördern die Reaktion. Das Reaktionsprodukt lagert sich auf der Zahnoberfläche ab („Deckschicht“; [13]). Die besondere Bedeutung der Deckschicht liegt darin, dass sie bei niedrigen pH-Werten wieder in Lösung geht und dabei Fluorid freigesetzt wird. Das freigesetzte Fluorid kann dann seinen remineralisierenden Effekt entfalten [14].

Die klinische Bedeutung der Kalziumfluoriddeckschicht liegt somit darin, dass sie einen Fluoridspeicher darstellt. Aus diesem Speicher wird genau an der Lokalisation und zu dem Zeitpunkt, zu dem Kohlenhydrate in der bakteriellen Plaque zu Säuren verstoffwechselt werden, Fluorid in niedriger Konzentration freigesetzt. Dieses Fluorid steht somit bedarfsgerecht zur Hemmung der Demineralisation und Förderung der Remineralisation an den Zahnhartsubstanzen zur Verfügung. Dies stellt einen entscheidenden Wirkungsansatz von Fluorid dar [9]. Da die Deckschicht aber nicht nur durch Säure gelöst wird, sondern auch durch mechanische Beanspruchungen reduziert wird, sind regelmäßige Fluoridapplikationen zum ständigen Wiederaufbau der Deckschicht erforderlich, z. B. mittels fluoridhaltiger Zahnpasta.

### Remineralisation

An der Zahnoberfläche liegt ein dynamisches Gleichgewicht von Lösung und Wiedereinlagerung von Mineral vor. Im subklinischen Bereich findet ein ständiger Wechsel von Phasen der De- und anschließender Remineralisation statt. Dabei wird Mineral zwischen dem Hydroxylapatit des Zahnschmelzes und der umgebenden flüssigen Phase in der interprismatischen Schmelzsubstanz, aber auch in Speichel und Plaque ausgetauscht. Allein durch seine Gegenwart beeinflusst Fluorid dieses dynamische Gleichgewicht zugunsten der Remineralisation [10]. Dabei wird sowohl die Demineralisation gehemmt als auch die Remineralisation gefördert [11].

### Aufnahme von Fluorid in den Zahnschmelz

Im Zusammenhang mit dem dynamischen De- und Remineralisationsgeschehen an der Schmelzoberfläche kann es zur Aufnahme von Fluorid in den Zahnschmelz kommen. Das Ausmaß der Fluoridaufnahme hängt vom pH-Wert, dem Mineralisationsgrad des Schmelzes und der einwirkenden Fluoridkonzentration ab. So werden in gesunden Zahnschmelz nur relativ geringe Mengen Fluorid eingelagert, die sich auf die oberen Mikrometer Zahnschmelz beschränken [15]. Dies ändert sich auch durch höhere Fluoridkonzentrationen nur wenig. Wenn hingegen demineralisierter Zahnschmelz vorliegt, kann Fluorid die Fehlstellen im Hydroxylapatitkristallgitter besetzen. Dadurch entstehen Mischkristalle von Hydroxyl- und Fluorapatit. Durch die Fluorideinlagerung in demineralisierte Schmelzareale, die bei Initialkaries vorliegen, wird die Kariesprogression verlangsamt oder gestoppt oder kann sogar soweit umgekehrt werden, dass ein Mineralgewinn resultiert. Damit wirkt Fluorid nicht nur primärpräventiv, sondern findet auch in der Sekundärprävention seinen Stellenwert.

Die mit Fluorid angereicherten remineralisierten Zahnoberflächen weisen eine erhöhte Widerstandsfähigkeit gegenüber kariogenen Angriffen auf [10, 11]. Dadurch ist fluoridierter, zwischenzeitlich demineralisierter Zahnschmelz besser gegen Säureangriffe gewappnet als der ursprüngliche gesunde Zahnschmelz [11]. Eine relevante Fluoridaufnahme in den Zahnschmelz erfolgt auch bei lokaler Fluoridapplikation kurz nach dem Zahndurchbruch, da die Zahnoberfläche zu diesem Zeitpunkt noch nicht vollständig mineralisiert ist. Im Gegensatz hierzu ist die Fluoridaufnahme vor dem Zahndurchbruch (präeruptiv) nach systemischer Fluoridgabe in den sich noch entwickelnden Zahn so gering, dass die Zähne klinisch nahezu ebenso säurelöslich sind wie nichtsystemisch fluoridierte Zähne [11].

### Effekte auf den bakteriellen Biofilm

Auch auf den bakteriellen Biofilm, der den Zähnen aufgelagert ist, kann Fluorid einwirken [11]. So können verschiedene Enzyme der bakteriellen Glykolyse gehemmt werden [16], wodurch Wachstum und Stoffwechsel oraler Mikroorganismen beeinträchtigt werden. Diese Stoffwechselbeeinflussung betrifft nur die Bakterien, nicht hingegen den Menschen, weil die menschlichen Körperzellen dank der Verdünnung des Fluorids in den Körperflüssigkeiten mit wesentlich geringeren Fluoriddosierungen als die oralen Bakterien konfrontiert werden. Zudem kann Fluorid mit der Anheftung von Mikroorganismen an der Zahnoberfläche interferieren [16, 17]. Insbesondere Zinn- und Aminfluoride zeigen diese Wirkung [18]. Dadurch kann die Entstehung einer kariesfördernden Plaque reduziert werden.

Bei den Fluoridkonzentrationen, die über Zahnpasten oder Spüllösungen in die Mundhöhle eingebracht werden, scheint der klinische Effekt der Bakterienstoffwechselhemmung nicht für einen erkennbaren Einfluss auf die Kariesentstehung auszureichen. Aktuelle Erklärungsmodelle der Kariesentstehung fokussieren auf pathologische Veränderungen in der bakteriellen Plaque, während Mundgesundheit mit stabilen bakteriellen Gemeinschaften innerhalb des Biofilms assoziiert ist. Unter dieser Sichtweise erhält der Einfluss von Fluorid auf den Biofilm eine neue, zusätzliche Bedeutung, denn die Symbiose der Mikroorganismen innerhalb des Biofilms scheint durch die Gegenwart von Fluorid gefördert zu werden [19].

### Einfluss auf Wurzelkaries

Über lange Zeit wurde der karieshemmende Einfluss von Fluorid vor allem mit Bezug auf Schmelzkaries bei Kindern und Jugendlichen untersucht. Die eingangs beschriebenen sich abzeichnenden Herausforderungen in Bezug auf die Prävention von Wurzeloberflächenkaries haben aber das Interesse an wissenschaftlicher und klinischer Bewertung einer Hemmung von Wurzelkaries durch Fluorid wachsen lassen. Hierbei ist die besondere Herausforderung, dass frei liegende Zahnwurzeln unter einer stoffwechselaktiven Plaque schneller eine Karies entwickeln können, als dies am Zahnschmelz der Fall ist. Mit ursächlich hierfür ist die höhere Säurelöslichkeit des Wurzeldentins [20]. Aus diesem Grund sind für eine erfolgreiche Kariesprävention am Wurzeldentin vergleichsweise höhere Fluoridkonzentrationen erforderlich [21]. Prinzipiell sind am Wurzeldentin aber die gleichen Fluoridwirkungsmechanismen aktiv wie am Zahnschmelz. Als zusätzliches Element der Karieshemmung durch Fluorid am Dentin wird diskutiert, dass Fluorid die im Zuge der Dentinkaries auftretende Degradation der organischen Kollagenmatrix hemmen könnte [22].

## Fluoridnebeneffekte

Wiederholt wurden gegenüber den zur Kariesprävention durchgeführten Fluoridanwendungen Vorhaltungen bezüglich deren Sicherheit gemacht. Dabei muss zwischen akuten und chronischen Effekten unterschieden werden. Akute toxische Effekte infolge Verschluckens großer Mengen fluoridhaltiger Mundpflegeprodukte auch durch Kinder sind allerdings maximal ein vorübergehendes Auftreten von Schwindelgefühl und Übelkeit. Die Seltenheit derartiger akuttoxischer Effekte mit in der Regel selbstlimitierendem Verlauf wird durch die jährlichen Berichte der American Association of Poison Control Centers über Meldungen unerwünschter Reaktionen nach Verschlucken von Zahnpasta belegt [23].

Verschiedene neuere Publikationen haben sich mit einer Reduktion der kognitiven Fähigkeiten von Kindern befasst, wenn diese oder ihre Mütter während der Schwangerschaft Fluorid aufgenommen hatten. Es ist herauszustellen, dass bei keinem dieser Berichte die zur Kariesprävention empfohlenen lokalen Fluoridierungsmaßnahmen Gegenstand der Bedenken sind [24]. Zudem weisen diese Studien methodische Mängel auf [24, 25]. Daher wird auch für systemische Fluoridierungsmaßnahmen darauf verwiesen, dass keine kognitiven Einschränkungen bei Kindern belegt sind [12].

Bei Kleinkindern und Kindern im Vorschulalter kann es jedoch zur Entstehung von Schmelzfluorosen kommen, wobei das Risiko mit höherer kontinuierlich erfolgender Fluoridaufnahme steigt. Fluorosen können nur während der Phase der Schmelzbildung entstehen; in anderen Altersgruppen kann dieser Nebeneffekt der Fluoridanwendung nicht ausgelöst werden. Bei den in Deutschland beobachteten Fluorosen handelt es sich zum übergroßen Teil um fragliche bis milde Ausprägungen, die keine Relevanz für die allgemeine Gesundheit der Kinder haben. Sie sind für die Zähne funktionell ohne Bedeutung und ästhetisch überwiegend unauffällig [26]. Dennoch sind die Erziehungspersonen eindringlich anzuhalten, die empfohlenen Zahnpastamengen einzuhalten.

## Fluorid zur Kariesprävention bei Kleinkindern und Kindern im Vorschulalter

Die verbreitetste Methode der Fluoridapplikation besteht in der Verwendung fluoridhaltiger Zahnpasten. Bereits für Kleinkinder und Kinder im Vorschulalter ist der kariespräventive Effekt von Fluoridzahnpasten nachgewiesen [27, 28]. Daher soll eine Zahnreinigung im häuslichen Rahmen ab dem Durchbruch des ersten Zahnes bis zum 2. Geburtstag zweimal täglich mit einer fluoridhaltigen Zahnpasta mit einem Fluoridgehalt von 1000 ppm erfolgen [29, 30]. Dabei ist die Menge der auf die speziell für Kleinkinder verfügbaren Zahnbürsten auf die Größe eines Reiskorns zu beschränken. Ab dem 2. bis zum 6. Geburtstag soll dann eine erbsengroße Menge der Kinderzahnpaste mit 1000 ppm Fluorid benutzt werden ([29, 30]; Tab. 2).

**Table 2:** 

Alter	Ppm Fluorid	Häufigkeit	Menge	Größe
Vom ersten Zahn an bis unter 2 Jahren	1000	Zweimal täglich	0,125 g	Reiskorngröße
2 bis 6 Jahre	1000^a^	Zweimal täglich	0,25 g	Erbsengröße

^a^Für Kinder im Alter von 2 bis 6 Jahren können je nach individuellem Kariesrisiko höhere Fluoridkonzentrationen in Betracht gezogen werden

Die in den aktuellen Empfehlungen zur Kariesprävention mit Fluorid bei Kleinkindern enthaltene Staffelung der empfohlenen Fluoridmenge ist im Hinblick auf eine möglichst effektive Kariesprävention bei gleichzeitiger toxikologischer Unbedenklichkeit (Vermeidung von Fluorosen) erfolgt. Für Kinder ab dem Alter von 2 Jahren kann darüber hinaus, z. B. in der Kindertagesstätte, ein drittes Mal mit einer erbsengroßen Menge einer 1000 ppm-Zahnpasta geputzt werden [31], ohne dass der Grenzwert des Tolerable Upper Intake Level (tolerierbare tägliche Aufnahmemenge) für Fluorid überschritten werden würde. Dieser wird mit einem Wert von 0,1 mg/kg Körpergewicht/Tag angegeben [32].

Auch Fluoridlacke sind für viele Alters- und Patientengruppen indiziert und können ab dem Kleinkindalter zur individuellen Kariesprävention eingesetzt werden. Ihre Anwendung erfolgt zumeist in der zahnärztlichen Praxis, darüber hinaus vielerorts aber auch in der Gruppenprophylaxe. Der Lack soll gezielt an Lokalisationen mit erhöhtem Kariesrisiko aufgetragen werden. Die meisten Fluoridlacke weisen eine deutlich höhere Fluoridkonzentration auf als Zahnpasten. Die toxikologische Unbedenklichkeit der Fluoridlackanwendung wurde aber auch für Kleinkinder nachgewiesen [33, 34].

In einer in Deutschland durchgeführten prospektiven Kohortenstudie konnte aufgezeigt werden, dass regelmäßige Fluoridlackapplikationen ab dem Kleinkindalter Kariesreduktionen bewirken [35]. In einer das internationale Schrifttum berücksichtigenden Metaanalyse wurde für das Milchgebiss ein durchschnittlicher kariespräventiver Effekt der Fluoridlackanwendung von ca. 37 % ermittelt, der wissenschaftlich mit hohem Evidenzgrad abgesichert ist [36].

Die kariesreduzierende Effektivität von Fluoridlacken ist auch für Kleinkinder und Kinder mit erhöhtem Kariesrisiko im Vorschulalter nachgewiesen worden [33, 34]. Bei Kindern mit erhöhtem Kariesrisiko wurde mit zunehmender Applikationshäufigkeit des Fluoridlackes eine steigende Karieshemmung aufgezeigt [33]. Die Applikationsfrequenz ist anhand des individuellen Kariesrisikos festzulegen.

Fluoridlacke sind nicht nur bezüglich der Entstehung von Karies primärpräventiv wirksam, sondern haben im Sinne der Sekundärprävention auch positive Effekte bei bereits vorhandener Initialkaries. Hier kann durch regelmäßige Fluoridlackapplikation, verbunden mit regelmäßiger Mundhygiene, häufig eine Kariesarretierung (Verhinderung weiterer Mineralverluste) erreicht werden [37]. Daher bietet der Einsatz von Fluoridlacken bei Vorliegen von Initialkaries, auch bei Kleinkindern, besondere Vorteile [34]. Hierbei werden geringe Lackmengen direkt auf die initialkariösen Schmelzareale appliziert.

Auch wenn die beschriebenen Fluoridierungsmaßnahmen bei Anwendung ab dem 1. Milchzahn wirkungsvoll der Kariesentstehung vorbeugen, müssen sie in ein generelles kariespräventives Konzept integriert werden. Dies beinhaltet die praktische Anleitung der Eltern zur Mundhygiene beim Kleinkind sowie Hinweise auf eine Ernährung mit begrenzter Zuckerzufuhr.

## Fluorid zur Kariesprävention bei Kindern, Jugendlichen und Erwachsenen

Der Großteil von Studien über die Bedeutung fluoridhaltiger Zahnpasten für die Kariesprävention im bleibenden Gebiss wurde an Kindern und Jugendlichen durchgeführt. Eine Cochrane-Metaanalyse von Studien in diesen Altersgruppen zeigte, dass durch Anwendung von Standardzahnpasten mit 1000–1450 ppm Fluorid eine Kariesreduktion um 22 % erzielt wird [5]. Diese Aussage ist mit hoher wissenschaftlicher Evidenz behaftet. In einer Aktualisierung dieser Übersichtsarbeit wurde für die Verwendung von Zahnpasta mit Fluoridgehalten von 1000 ppm oder 1450 ppm über ca. 3 Jahre eine mittlere Reduktion der Anzahl von Karies betroffener Zähne (DMFT-Werte) um 0,26 bzw. 0,37 Zähne errechnet [3].

Für die professionell angewendeten Fluoridlacke wurde im bleibenden Gebiss in einer Übersichtsarbeit eine mittlere Kariesreduktionrate von 43 % gefunden, die mit hohem Evidenzgrad untermauert ist [36]. Die Fluoridlackapplikation wird in risikoabhängigen Zeitintervallen zwei- bis viermal jährlich, in Einzelfällen auch öfter, empfohlen.

Für Kinder ab dem Alter von 6 Jahren und für Jugendliche stehen weitere Fluoridierungsmaßnahmen wie Gelees oder Mundspüllösungen zur Verfügung. Ihre Anwendung kann insbesondere bei erhöhtem Kariesrisiko indiziert sein. 2 Cochrane-Reviews haben, bezogen auf den DMFT-Wert, für Gelees mittlere Kariesreduktionsraten von 32 % bzw. für Spüllösungen von 23 % errechnet [38, 39]. Die Effekte wurden jeweils unabhängig vom Fluoridgehalt der verwendeten Zahnpasta gefunden.

In der Erwachsenenbevölkerung in Europa sind ebenfalls deutliche Rückgänge der Karieslast dokumentiert worden [40]. Auch wenn Studien über den karieshemmenden Effekt lokaler Fluoridapplikationen in dieser Altersgruppe selten durchgeführt wurden, kann aus der vorhandenen Datenlage errechnet werden, dass Fluorid bei Erwachsenen jeden Alters Karies an Kronen und Wurzeln hemmt [41, 42]. In älteren Studien wurde zudem der Nachweis geführt, dass Zahnpasten mit Fluorid bei Personen im Alter von 18–93 Jahren karieshemmende Effekte aufweisen [3]. Eine weitere Metaanalyse zeigt, dass Initialkaries an oralen und vestibulären Glattflächen mittels höher konzentrierter Fluoridpräparate wie Gelen und Lacken arretiert oder remineralisiert werden kann [43].

## Fluorid zur Kariesprävention bei Senioren

Die oben geschilderten Erfolge der Oralprävention gehen mit einer im Vergleich zu früheren Jahren höheren Anzahl eigener Zähne bei der älteren Bevölkerung einher. Dies bedeutet aber auch, dass die Erfordernisse der Kariesprävention sich auf spätere Lebensabschnitte hin erweitern. Im Grundsatz unterscheiden sich hier die Maßnahmen der Kariesprävention für Senioren, die selbstständig ihren Alltag bestreiten, nicht von den Maßnahmen in jüngeren Lebensjahren. So gilt auch für spätere Lebensabschnitte die Empfehlung, sich mindestens zweimal täglich die Zähne mit fluoridhaltiger Zahnpasta zu putzen. Oft ist aber die Fähigkeit zu einem angemessenen Zahnputzverhalten bei manchen Senioren nicht mehr hinreichend gegeben. So können Geschicklichkeit und Sehkraft im höheren Alter soweit nachlassen, dass mechanische Mundhygienemaßnahmen nicht mehr erfolgreich umgesetzt werden können [44]. In dieser Situation werden verstärkt zusätzliche Maßnahmen der Kariesprävention erforderlich. Auch für erkrankte oder pflegebedürftige bezahnte Personen müssen zusätzliche oder speziell angepasste Präventionsmaßnahmen ergriffen werden. Hier erhalten auch Fluoridierungsmaßnahmen eine weitere Bedeutung.

In der Altersgruppe der Senioren wurden Studien über die Prävention von Kronenkaries durch lokale Fluoridapplikationen nur in begrenzter Anzahl durchgeführt [45]. Unwidersprochen wird aber auch für Senioren der Applikation von Fluoriden mittels Zahnpasten hohe Bedeutung für die Prävention der Karies an Zahnkronen zuerkannt [41, 44]. Angesichts des mit zunehmendem Alter häufig erhöhten Kariesrisikos und der Konzentrations-Wirkungs-Beziehung zwischen Fluorid und Karieshemmung gilt die Anwendung einer hochkonzentrierten Fluoridzahnpasta mit 5000 ppm Fluorid als besonders effektiv [44, 46].

Eine größere Anzahl an Studien hat die Prävention und Sekundärprävention von Wurzeloberflächenkaries bei Senioren zum Inhalt [45, 47]. Übereinstimmend wird die Karieshemmung auch an der Zahnwurzel durch Applikation von Fluoridpräparaten beschrieben. Sofern das Kariesrisiko nicht erhöht ist, können fluoridhaltige Zahnpasten sowie fluoridhaltige Mundspüllösungen ausreichend präventive Wirkung entfalten [41, 47]. Besonders effektiv sind jedoch höher konzentrierte Fluoridzubereitungen [45]. Dies gilt für Zahnpasten mit bis zu 5000 ppm Fluoridgehalt, die in Deutschland als Arzneimittel verschreibungspflichtig sind, und für Fluoridlacke [45]. Bei Senioren mit erhöhtem Wurzelkariesrisiko ist eine engere Applikationsfrequenz dieser Lacke indiziert. Diese Empfehlungen gelten auch für pflegebedürftige Senioren [48, 49]. Schließlich konnte bei Senioren mit oberflächlicher Wurzelkaries auch der sekundärpräventive Effekt von hochkonzentrierten Zahnpasten und Fluoridlacken nachgewiesen werden [50, 51].

## Fazit

- Die lokale Anwendung von Fluoridpräparaten an der Zahnoberfläche gilt als zentrale Ursache für den in fast allen Altersgruppen festgestellten Rückgang der Kariesprävalenz und Karieserfahrung.
- Im Milchgebiss, bei älteren Personengruppen und bei Patienten mit erhöhtem Kariesrisiko besteht Bedarf nach intensivierten Fluoridierungsmaßnahmen.
- Fluorid erzielt die karieshemmende Wirkung durch Bildung einer Fluoridspeicherschicht auf der Zahnoberfläche, Remineralisationsvorgänge und antibakterielle Effekte, die zusammen an den Zahnhartsubstanzen und im Biofilm ansetzen.
- Es liegt eine Konzentrations-Wirkungs-Beziehung vor, bei der höhere lokal einwirkende Fluoridkonzentrationen eine gesteigerte Karieshemmung bewirken.
- Fluoridhaltige Zahnpasten sollen ab dem ersten Milchzahn verwendet werden.
- Es bestehen keine allgemeintoxikologischen Bedenken gegenüber der lokalen Fluoridanwendung.
- Bei Kleinkindern ist die Menge der verwendeten Zahnpasta auf die empfohlenen Volumina zu beschränken, um das Risiko der Entstehung von Fluorosen zu begrenzen.
- Fluoridlacke weisen bei erhöhtem Kariesrisiko sowie bei bereits vorhandenen Demineralisationen besondere Vorteile auf.
- Am Wurzeldentin können durch Anwendung von hochkonzentrierter Fluoridzahnpasta, fluoridhaltigen Gelen oder Fluoridlacken primär- und sekundärpräventive Effekt erzielt werden.
- Die Fluoridanwendung muss Bestandteil eines oralpräventiven Gesamtkonzeptes sein, in dem Mundhygienemaßnahmen zur Reduktion des kariogenen Biofilms sowie eine ausgewogene Ernährungsweise mit verringerter Saccharosezufuhr ebensolchen Stellenwert haben.
